# Preoperative Master’s double two-step test may predict survival after lobectomy in patients with lung cancer

**DOI:** 10.1186/s13019-022-01850-6

**Published:** 2022-05-03

**Authors:** Satoshi Shiono, Makoto Endo, Kenta Nakahashi, Marina Nakatsuka

**Affiliations:** Department of Thoracic Surgery, Yamagata Prefectural Central Hospital, 1800, Oazaaoyagi, Yamagata, 990-2292 Japan

**Keywords:** Master’s double two-step test, Lung cancer, Thoracic surgery

## Abstract

**Background:**

The Master’s double two-step test (MDT), which is used to screen for coronary heart disease, is difficult for physically impaired patients to complete. The purpose of this study was to clarify the relationship between the results of the MDT and prognosis after lung cancer surgery.

**Methods:**

Between May 2004 and September 2019, 1,434 patients underwent complete resection for lung cancer at our hospital. Among them, 418 with pathological stage I disease who underwent lobectomy were evaluated. We defined patients who could accomplish the MDT as the complete MDT group and those who could not as the incomplete MDT group. Patients who could not perform the MDT due to physical problems were included in the incomplete MDT group. We explored the prognostic impact of the MDT results in these patients.

**Results:**

Fifty-three patients (12.7%) were in the incomplete MDT group; compared with the complete MDT group, they were older and had poorer performance status and respiratory function. However, the incidence of postoperative complications and 90-day mortality did not differ significantly between groups. Multivariate analyses revealed that age (*p* < 0.001), Charlson comorbidity index (*p* = 0.013), incomplete MDT (*p* = 0.049) and carcinoembryonic antigen (CEA) level (*p* = 0.003) were prognostic factors for worse overall survival; age (*p* < 0.001) and incomplete MDT (*p* = 0.022) were prognostic factors for worse non-cancer-specific survival.

**Conclusions:**

Although incomplete MDT was not associated with postoperative complications, 90-day mortality or cancer-specific survival, MDT results may be significantly associated with non-cancer-specific survival.

## Background

Lung cancer is the leading cause of cancer death worldwide [1]. However, with the advent of chest computed tomography (CT), small-sized lung cancer is now more likely to be detected, and outcomes for patients with lung cancer have improved. For lung cancer surgery to be performed safely, preoperative assessment is essential [2, 3]. Among the preoperative tests, cardiopulmonary exercise testing has been recognized as a diagnostic tool that can evaluate the physical reserve of the heart, lungs and skeletal muscles. Low peak oxygen uptake evaluated with cardiopulmonary exercise testing has been demonstrated as a risk factor for cardiovascular and pulmonary complications [4]. However, such testing is not always performed easily. As alternatives, the 6-min walk test (6MWT) [5, 6], stair-climbing test [7], and shuttle walk test [8] are low technology exercise tests that can evaluate patient tolerance and the perioperative risk of lung cancer surgery. These tests can also predict morbidity and mortality after lung cancer surgery [2, 4–8].

Since the Master’s double two-step test (MDT) is an exercise electrocardiogram test to screen for coronary heart disease [9, 10], we routinely perform MDT prior to lung cancer surgery. The MDT corresponds to approximately 6 metabolic equivalents of task (METs). As patients with lung cancer tend to have smoking-induced cardiopulmonary comorbidity, preoperative assessment of cardiac comorbidity is needed. The MDT can detect the presence of coronary heart disease. On the other hand, because the MDT is a type of stair-climbing test, it reflects the exercise tolerance and physical activity of patients planning to undergo surgery, and it is possible that the MDT is associated with morbidity or mortality after lung surgery, like other “low-technology” tests.

While these tests evaluate physical activities, physical activities are recognized to be associated with survival after lung cancer treatment [7]. Brunelli et al. revealed that the 5-year survival of patients who climbed > 18 m in the preoperative stair-climbing test was longer than that of patients who climbed < 18 m [7]. Preoperative physical activity is considered a prognostic factor for outcomes after lung cancer surgery.

From this point of view, we considered that the results of the MDT could predict patient survival after lung cancer surgery. The MDT could be a surrogate for the prognosis of patients who undergo lung cancer surgery. The purpose of this study was to clarify the relationship between the results of the MDT and prognosis after lung cancer surgery.

## Patients and methods

This was a retrospective cohort study using prospectively collected patient data from the patient database of our institute. The ethics committees of our institute approved this study (No. 134, January 14, 2020) and waived the need for informed consent from patients because all patient data remained anonymous.

### Database

We used a database of patients undergoing surgery for non-small cell lung cancer (NSCLC), constructed from the following data: (1) patient demographics (age, gender, smoking status, body height, body weight, body mass index, results of the MDT, tumor markers, comorbidities, and pulmonary function tests), (2) TNM classification (8^th^ edition) of lung cancer, (3) surgical procedures, (4) final pathological findings, (5) complications, and (6) outcomes and date of event (readmission, site of recurrence, death, and follow-up). The data were reviewed weekly by the authors.

Preoperative examination consisted of physical examination, blood chemistry, blood gas analysis, MDT, pulmonary function, chest X-ray, chest and brain contrast-enhanced computed tomography (CT), and positron emission tomography (PET)/CT. The Charlson comorbidity index (CCI) was calculated from the status of comorbidities.

## Master’s double two-step test (MDT)

The MDT was carried out in accordance with the standard procedure [9] within 1 month of surgery. In addition, the MDT was performed before preoperative rehabilitation was started. During the test, patients were asked to climb the 2 steps, which were standardized in size (height of 23 cm, depth of 25 cm, width of approximately 50 cm), rhythmically for 3 min. All patients were accompanied by a medical technician. After climbing, patients were monitored by electrocardiogram every minute for up to 5 min. The MDT results were evaluated by a cardiologist. Patients who were positive or suspicious for an ischemic change on the MDT were referred to the cardiology department for consultation. Patients who could not climb the steps due to physical disabilities or cardiac problems were exempt from the MDT. During climbing, if patients experienced chest pain, dyspnea, leg fatigue or leg pain, the test was stopped based on the judgment of a medical technician.

In this study, we divided the patients into 2 groups. Regardless of the results of the MDT, we defined patients who could accomplish the MDT as the complete MDT group and those who could not as the incomplete MDT group. Patients who could not perform the MDT due to physical problems were included in the incomplete MDT group.

### Study objectives

**Fig. 1:**
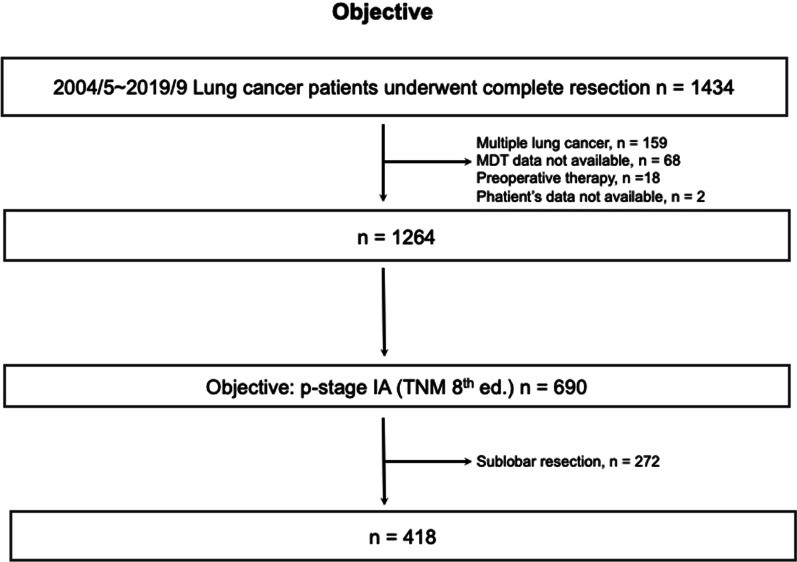
Flow of patients through the study

From May 2004 to September 2019, 1,434 patients underwent complete resection for lung cancer. Patients were excluded for the following reasons: multiple lung cancers (n = 159), MDT data not available (n = 68), preoperative treatment (n = 18) or patient data not available (n = 2). Since patients who underwent wedge resection or segmentectomy tended to have poor performance status, impaired respiratory function or comorbidities, 272 patients who underwent segmentectomy or wedge resection were excluded, and 418 patients with p-stage IA who underwent lobectomy were evaluated (Fig. 1).

### Surgical procedure

Lobectomies carried out via an open thoracotomy were standard procedures for NSCLC at our institution. The posterolateral thoracotomy incision that spared the serratus anterior muscle was approximately 8 cm. The rib that was cut varied according to the patient.

### Postoperative complications

According to the Clavien-Dindo classification system [11, 12], postoperative complications of grade ≥ II were defined as postoperative complications in the present study. Pulmonary complications included prolonged air leakage, hypoxemia, acute respiratory failure, pneumonia, atelectasis, empyema, and pleural effusion. Cardiovascular complications included arrhythmia and ischemic heart disease. Other complications included wound infection and urinary tract infection.

### Surveillance and evaluation of recurrence

The postoperative surveillance schedule consisted of outpatient clinic visits 1 or 2 weeks after surgery and every month thereafter for up to 3 months. Basically, postoperative surveillance was performed by thoracic surgeons every 6 months for 5 years. Regular outpatient clinic check-ups, including physical examination, blood chemistry including serum tumor markers, and chest X-ray or CT were performed two times a year for 5 years depending on the stage of the patients. If signs of recurrence were found, further visits were recommended to the patients. During the follow-up period, if recurrence was suspected, these radiological studies, as well as PET/CT, bone scintigraphy, and/or brain magnetic resonance imaging were performed.

The date of histologic confirmation or radiologic diagnosis of recurrence was defined as the date of recurrence. Recurrence was determined by the cancer treatment board, which included a thoracic surgeon, a physician, and a radiologist.

### Statistical methods

Primary endpoint was overall survival and secondary endpoint was cancer-specific survival and non-cancer specific survival. Patient background and clinical characteristics were compared with the chi-square test or Fisher exact test. Analysis of variance was used to compare continuous variables in patients who did and did not complete the MDT. Survival was analyzed with the Kaplan–Meier method. Cox proportional hazards modeling was used in univariate and multivariate analyses to evaluate prognostic factors for survival. Variables with *p* values of less than 0.05 in univariate analysis were used in a multivariate Cox’s proportional hazards model.

Overall survival was defined as the time from the date of surgery to the date of death from any cause or censored at the date of the patient’s last hospital visit. Cancer-specific survival was defined as the time from the date of surgery to the date of death from lung cancer, and non-lung cancer causes of death were censored. Non-cancer-specific survival was defined as the time from the date of surgery to the date of non-lung cancer death, and lung cancer causes of death were censored. If the cause of death could not be determined, the deaths of patients with recurrence were regarded as cancer-specific deaths. Survival differences were assessed using the log-rank test. Data were analyzed with version 11 of the JMP software package (SAS Institute Inc., Cary, NC, USA). A *p* value < 0.05 was considered to indicate statistical significance.

## Results

Regarding the results of the MDT, 365 patients (87.3%) completed and 53 (12.7%) patients did not complete the MDT. The median follow-up time was 60 months. Patient demographics in the 2 cohorts are summarized in Table 1. By the appearance of ischemic change, we classified into negative, borderline, and positive. Among the 365 patients who completed the MDT, 292 were negative, 51 were borderline, and 22 were positive. Among the 53 patients who did not complete the MDT, the reasons were as follows: weakness of legs, n = 42; shortness of breath, n = 5; fatigue, n = 3; and cardiac problems, n = 3 (coronary artery disease 2, atrial fibrillation 1). The groups differed in terms of patient characteristics (Table 1). Significantly higher proportions of elderly patients and patients with impaired pulmonary function were present in the incomplete MDT group. In terms of tumor biological characteristics, non-adenocarcinoma, advanced stage, pathological invasiveness and higher standardized max value on PET/CT were dominant in the incomplete MDT group.

**Table 1:** Patient characteristics by the results of the Master’s double two-step test

Factors	Complete MDT, n = 365	Incomplete MDT, n = 53	*p* value
n (%) or median (IQR)			
*Gender, n (%)*			
Male	193 (52.9)	31 (58.5)	0.443
Female	172 (47.1)	22 (41.5)	
Age (IQR), years	68 (61–73)	75 (68–81)	< 0.001
*Smoking status, n (%)*			
Smoker	200 (54.8)	32 (60.4)	0.443
Non-smoker	165 (45.2)	21 (39.6)	
Charlson comorbidity index, median (IQR)	0 (0–2)	0 (0–2)	0.076
*Performance status, n (%)*			
0	363 (99.7)	51 (96.2)	0.044
1, 2	1 (0.3)	2 (3.8)	
CEA, median (IQR), (ng/ml)	2.5 (1.6–3.9)	2.9 (2.1–4.0)	0.663
SUV max, median (IQR)	2.9 (1.5–5.2)	3.9 (2.2–9.0)	< 0.001
FVC, median (IQR), L	3.17 (2.66–3.75)	2.74 (2.30–3.67)	0.013
%FVC, median (IQR), %	108.7 (99.2–119.5)	104.0 (92.1–112.5)	0.006
FEV1 median (IQR), L	2.29 (1.92–2.76)	1.89 (1.62–2.42)	< 0.001
FEV1% median (IQR), %	74.7 (67.7–79.8)	69.6 (65.9–75.2)	0.009
Body mass index, median (IQR), kg/m^2^	22.8 (20.8–24.8)	21.6 (19.6–24.8)	0.458
PaO_2_ median (IQR), mmHg	84.4 (77.4–91.0)	81.6 (75.0–90.4)	0.091
PaCO_2_ median (IQR), mmHg	39.3 (37.2–42.0)	38.1 (35.7–40.5)	0.007
*Histological type, n (%)*			0.005
Adenocarcinoma	299 (81.9)	34 (64.2)	
Non-adenocarcinoma	66 (18.1)	85 (20.3)	
*Pathological stage, n (%)*			
0	22 (6.0)	1 (1.9)	0.045
IA1	140 (38.4)	16 (30.2)	
IA2	126 (34.5)	16 (30.2)	
IA3	77 (21.1)	20 (37.7)	
Invasive component size, median (IQR), cm	1.2 (0.5–1.8)	1.7 (0.8–2.4)	0.011
Lymphatic invasion positive, n (%)	12 (3.3)	2 (3.8)	0.694
Vascular invasion positive, n (%)	18 (4.9)	1 (1.9)	0.490
STAS positive, n (%)	57 (15.8)	12 (22.6)	0.224

MDT: Master’s double two-step test, IQR: interquartile range, CEA: carcinoembryonic antigen, FVC: forced vital capacity, FEV1: forced expiratory volume in 1 s, FEV1%: forced expiratory volume as a percentage of forced vital capacity, STAS: spread through air spaces, SUV: standardized uptake value

Table 2 shows the short-term surgical outcomes after lobectomy. Length of postoperative stay and frequency of postoperative complications ≥ Grade 2 by the Clavien-Dindo classification did not differ between the complete and incomplete MDT groups. There was no perioperative death within 30 days. There was 1 death in each group within 90 days of surgery (Table 2). The details of the postoperative complications are shown in Table 3. Arrhythmia was the most common complication. During the study period, 15 patients died from lung cancer, 16 died from other cancers and 31 died from non-cancer death. Between both groups, the proportion of non-cancer deaths was significantly higher in the incomplete MDT group (Table 4). Regarding with the death cause of incomplete MDT group, the causes were as follows: heart disease, n = 3, pneumonia, n = 3; accident n = 1; amyotrophic lateral sclerosis, n = 1; and unknown, n = 3.

**Table 2:** Surgical outcomes by the results of the Master’s double two-step test

Outcomes	Complete MDT, n = 365	Incomplete MDT, n = 53	*p* value
Median (IQR) postoperative stay, days	6 (4–9)	5 (4–8)	0.552
Postoperative complications, n (%)	51 (14.0)	11 (20.8)	0.213
Mortality within 30 days, n (%)	0 (0)	0 (0)	–
Mortality within 90 days, n (%)	1 (0.3)	1 (1.9)	0.239

MDT: Master’s double two-step test, IQR: interquartile range

**Table 3:** Postoperative complications by the results of the Master’s double two-step test in detail

	Complete MDT, n = 365, n (%)	Incomplete MDT, n = 53, n (%)
Postoperative complications	51 (14.0)	11 (20.8)
Arrhythmia	18	4
Prolonged air leakage	11	2
Pleural effusion	5	0
Delirium	3	2
Wound infection	3	0
Hypoxia	3	3
Empyema	2	1
Postoperative bleeding	2	0
Urinary tract infection	2	0
Pneumonia	1	2
Chylothorax	1	0
Pulmonary vein thrombosis	1	0
Atelectasis	1	0
Hoarseness	1	0

MDT: Master’s double two-step test

**Table 4:** Cause of death in the complete and incomplete MDT groups

Outcome	Complete MDT, n = 365, n (%)	Incomplete MDT, n = 53, n (%)	*p* value
Alive	318 (87.1)	38 (71.7)	0.006
Lung cancer death	13 (3.6)	2 (3.8)	0.939
Other cancer death	14 (3.8)	2 (3.8)	0.983
Non-cancer death	20 (5.5)	11 (20.8)	< 0.001

MDT: Master’s double two-step test

In terms of the prognostic factors for overall survival, Table 5 shows the results of univariate and multivariate analyses. Multivariate analysis for overall survival showed that higher age, higher CCI, incomplete MDT, and higher serum CEA level were significantly associated with worse prognosis. Table 6 shows the prognostic cancer-specific factors. In multivariate analysis, higher age and CEA level remained prognostic factors. Regarding non-cancer-specific survival (Table 7), multivariate analysis showed that higher age and incomplete MDT were significantly associated with worse prognosis.

**Table 5:** Prognostic factors for overall survival

Variable	Univariate analysis	*p* value	Multivariate analysis	*p* value
	Risk ratio (95% CI)		Risk ratio (95% CI)	
Male	2.55 (1.47–4.65)	< 0.001	1.15 (0.39–3.44)	0.807
Age	1.10 (1.06–1.14)	< 0.001	1.08 (1.03–1.12)	< 0.001
PS 1, 2	6.58 (1.59–18.1)	0.014	1.59 (0.34–5.29)	0.514
Smoking history	2.33 (1.35–4.19)	0.002	1.49 (0.51–4.54)	0.476
CCI	1.34 (1.20–1.46)	< 0.001	1.20 (1.04–1.35)	0.013
Incomplete MDT	3.48 (1.92–6.32)	< 0.001	2.05 (1.00–4.02)	0.049
CEA level	1.07 (1.04–1.10)	< 0.001	1.06 (1.03–1.09)	0.003
SUV max	1.08(1.03–1.12)	0.002	0.99 (0.92–1.07)	0.844
Invasive component size	1.07 (1.04–1.11)	< 0.001	1.04 (0.99–1.10)	0.083
Non-adenocarcinoma	2.48 (1.47–4.11)	< 0.001	0.91 (0.41–1.99)	0.811
Lymphatic invasion	1.17 (0.35–2.88)	0.771		
Vascular invasion	1.68 (0.41–4.57)	0.420		
STAS	1.08 (0.53–2.00)	0.814		

CI: confidence interval, PS: performance status, CCI: Charlson comorbidity index, MDT: Master’s double two-step test, CEA: carcinoembryonic antigen, SUV: standardized uptake value, STAS: spread through air spaces

**Table 6:** Prognostic factors for cancer-specific survival

Variable	Univariate analysis	*p* value	Multivariate analysis	*p* value
	Risk ratio (95% CI)		Risk ratio (95% CI)	
Male	2.60 (0.89–9.39)	0.083		
Age	1.07 (1.01–1.15)	0.026	1.09 (1.00–1.19)	0.045
PS 1, 2	–	0.600		
Smoking history	1.81 (0.64–5.82)	0.266		
CCI	1.24 (0.94–1.50)	0.113		
Incomplete MDT	1.43 (0.22–5.25)	0.657		
CEA level	1.15 (1.07–1.28)	< 0.001	1.10 (1.02–1.25)	0.008
SUV max	1.14 (1.05–1.22)	0.004	1.05 (0.91–1.19)	0.475
Invasive component size	1.11 (1.04–1.20)	< 0.001	1.03 (0.92–1.15)	0.550
Non-adenocarcinoma	4.71 (1.69–13.4)	0.004	1.44 (0.25–8.61)	0.681
Lymphatic invasion	8.35 (2.29–24.7)	0.003	4.15 (0.60–20.0)	0.134
Vascular invasion	7.31 (1.65–23.5)	0.013	2.62 (0.37–12.5)	0.297
STAS	1.99 (0.55–5.83)	0.267		

CI: confidence interval, PS: performance status, CCI: Charlson comorbidity index, MDT: Master’s double two-step test, CEA: carcinoembryonic antigen, SUV: standardized uptake value, STAS: spread through air spaces

**Table 7:** Prognostic factors for non-cancer-specific survival

Variable	Univariate analysis	*p* value	Multivariate analysis	*p* value
	Risk ratio (95% CI)		Risk ratio (95% CI)	
Male	4.16 (1.71–12.4)	0.001	1.32 (0.25–7.79)	0.764
Age	1.15 (1.09–1.22)	< 0.001	1.13 (1.07–1.21)	< 0.001
PS 1, 2	17.0 (3.95–50.7)	0.001	3.22 (0.67–11.4)	0.129
Smoking history	4.23 (1.75–12.6)	0.001	2.82 (0.52–18.1)	0.251
CCI	1.25 (1.02–1.45)	0.035	0.95 (0.71–1.18)	0.662
Incomplete MDT	6.36 (2.75–14.0)	< 0.001	3.08 (1.18–7.75)	0.022
CEA level	1.05 (0.97–1.09)	0.143		
SUV max	1.07(1.01–1.14)	0.036	0.96 (0.85–1.07)	0.467
Invasive component size	1.08 (1.04–1.13)	< 0.001	1.06 (0.99–1.13)	0.077
Non-adenocarcinoma	2.93 (1.39–6.03)	0.006	0.78 (0.26–2.35)	0.649
Lymphatic invasion	–	0.045		
Vascular invasion	–	0.201		
STAS	1.18 (0.44–2.74)	0.717		

CI: confidence interval, PS: performance status, CCI: Charlson comorbidity index, MDT: Master’s double two-step test, CEA: carcinoembryonic antigen, SUV: standardized uptake value, STAS: spread through air spaces

**Fig. 2:**
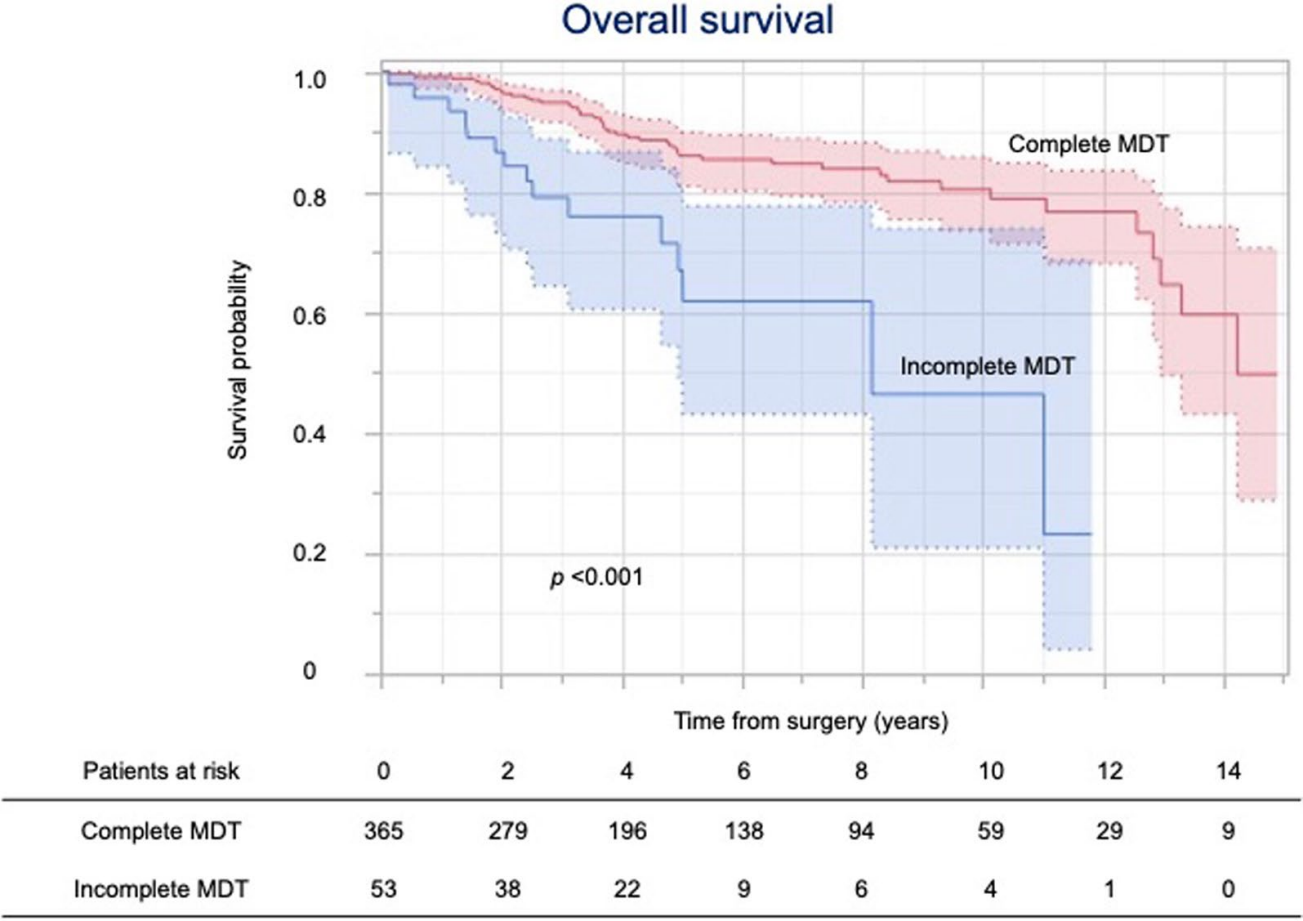
Kaplan–Meier curves for overall survival

**Fig. 3:**
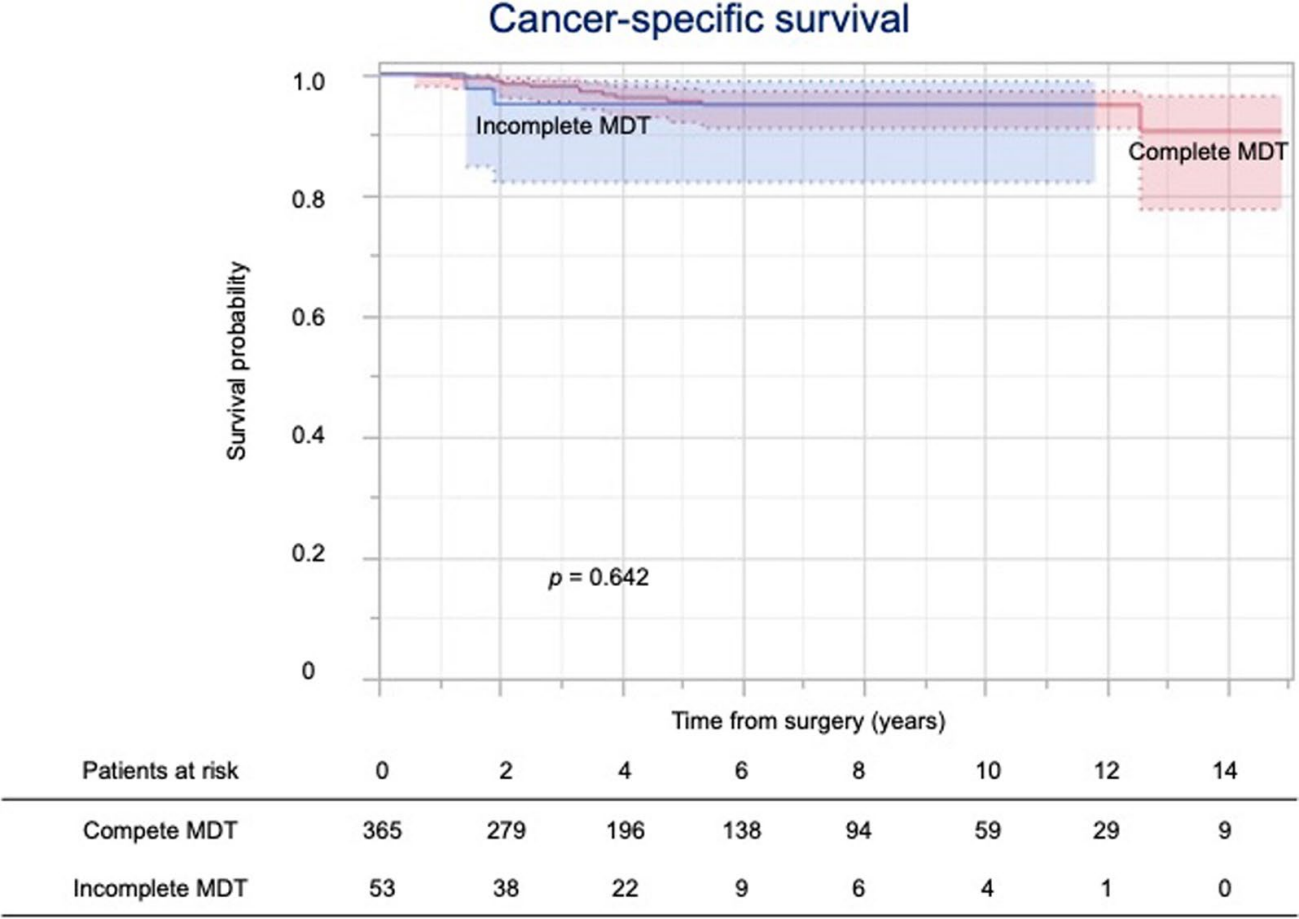
Kaplan–Meier curves for cancer-specific survival

**Fig. 4:**
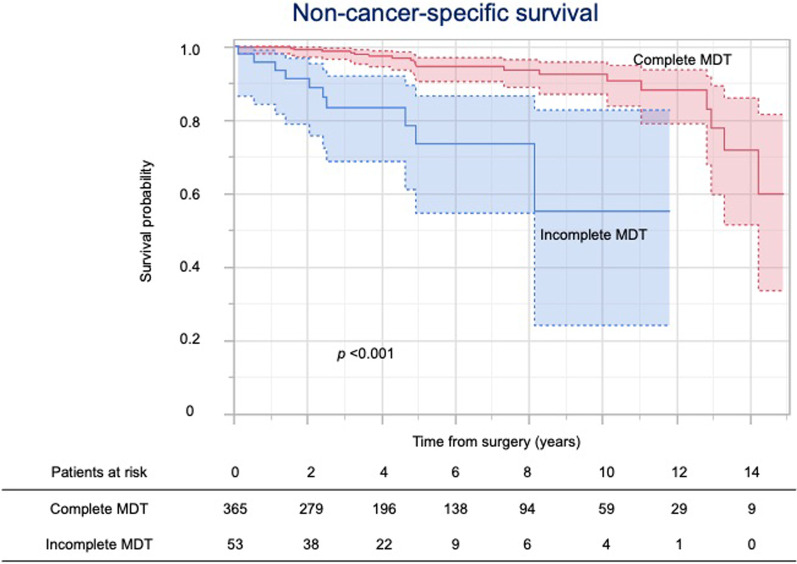
Kaplan–Meier curves for non-cancer-specific survival

Figure 2 shows the overall survival by completion of the MDT. The 5-year overall survival rates of patients with complete and incomplete MDT were 86.0% and 67.0%, respectively. While the 5-year cancer-specific survival rates of patients with complete and incomplete MDT were 95.5% and 95.0%, respectively (Fig. 3), the 5-year non-cancer-specific survival rates were 94.5% and 73.5%, respectively (Fig. 4).

## Discussion

According to the annual report of the Japanese Association for Thoracic Surgery, the proportion of patients aged ≥ 70 years who underwent lung cancer surgery was 24,799 of the 44,140 total cases (56.2%). Moreover, 5,779 of the 44,140 total cases (13.1%) who underwent lung cancer surgery were aged ≥ 80 years [13]. For elderly patients, physical activity has an important role in the safety of lung cancer surgery. Therefore, meticulous preoperative management is mandatory [2, 3].

Poor physical activity is a potential risk factor for poor outcomes of lung cancer surgery [4–8], and frailty is considered predictive of postoperative complications [14]. To evaluate patient frailty, Makary et al. advocated the frailty score, which is composed of weight loss, decreased grip strength, exhaustion, low physical activity and slowed walking speed [14]. Another method to evaluate patient physical activity, the Eastern Cooperative Oncology Group performance status [15], is a well-known prognostic factor and is commonly used for decision-making regarding lung cancer surgery. However, Dajczman et al. reported that patient-rated performance status was not identical to physician-related performance status in 54% of all cases [16]. For accurate preoperative assessment of patient physical activity, objective assessment is required. ERS/ESTS clinical guidelines for patients planning to have lung cancer surgery emphasize the importance and role of exercise tests [2]. Although there was an argument of the result of low technology exercise tests [17], the 6MWT [5, 6] can predict patient tolerance and perioperative risk of lung cancer surgery. Regarding the shuttle walk test, an inability to walk 250 m was associated with a very high incidence of postoperative cardiopulmonary complications (44%) [8].

The MDT is a screening test for ischemic heart disease. Since the MDT could reflect the exercise tolerance and physical activity of patients, we assumed that it would be closely associated with short- and long-term outcomes after lung cancer surgery. The present study demonstrated that the MDT could not predict postoperative complications and short-term survival, and it might overestimate the surgical risk. We suggest that preoperative rehabilitation could reduce postoperative complications.

The present study revealed that the MDT could be a significant prognostic factor for overall survival after lung cancer surgery. A systematic review found that patients with cancer who have more positive exercise behaviors tended to have a lower risk of all-cause death [18]. To date, few studies have shown an association between exercise tolerance and prognosis after lung cancer surgery. Brunelli et al. investigated the prognostic impact of the stair-climbing test in patients who underwent pulmonary resection for pathological stage I NSCLC and revealed that the 5-year overall survival of patients who climbed > 18 m in a preoperative stair-climbing test was longer than that of patients who climbed < 18 m [7]. They also revealed that the cancer-specific survival of patients who climbed < 18 m was worse than that of patients who climbed > 18 m. Our study suggests that patients who could not complete the MDT tended to have worse overall survival, and those patients are more likely to die from diseases other than cancer. Kanzaki et al. revealed that age (≥ 70), male sex, body mass index (< 18.5 kg/m^2^), postoperative complications and low preoperative % forced expiratory volume 1 (< 80) were risk factors for postoperative non-cancer death after surgery in patients with stage I NSCLC [19]. Their results are reflected by the frailty of the patients who could not complete the MDT. There is a possible explanation for the present results. It is inferred that if patients who could complete the MDT exercised regularly, that could reduce the risk of developing comorbid conditions, such as heart disease, hypertension, diabetes, and cerebrovascular disease.

The strength of this study is as follows: Because the MDT is a low technology test, it can check patient status easily, including the evaluation of ischemic heart disease. Given its convenience in clinical practice, the MDT could be pivotal for choosing surgical procedures or excluding patients who are not fit for surgery. We postulate that easier tests and uncomplicated algorithms are more commonly reproduced in daily practice. The results of our study suggest that improvement in physical activity could lead to better survival. Some trials have aimed to improve the physical activity of patients who are planning to undergo lung cancer surgery [20, 21].

The present study has several limitations. First, it was a retrospective study carried out at a single institution. To obtain robust evidence, a multi-institutional cohort study based on the standard evaluation criteria is needed. Second, judgment of complete or incomplete MDT was based on the discretion of each medical technologist, which contributes potential biases. Third, for patients who could not walk well, the MDT might have a risk of falling and injury. According to a study of sarcopenia and the 6MWT [22], the proportion of patients ≥ 75 years old was 24% among those who could not walk a long distance. Therefore, roughly one quarter of the patients might not be able to complete the MDT. Finally, since the MDT does not measure the SpO_2_%, we could not show the role of desaturation during exercise. However, while desaturation during exercise is considered to be associated with postoperative complications, there are several controversial results [8, 17].

In conclusion, patients who could not complete the MDT were older and had poorer performance status and respiratory function than patients who could complete it. The results of the MDT were not associated with postoperative complications. Although the MDT cannot evaluate all patients who plan to have lung cancer surgery, the results of the MDT could be useful for identifying patients with potentially poor prognosis, especially for overall survival.

## Data Availability

The datasets used and/or analysed during the current study are available from the corresponding author on reasonable request.

## Abbreviations

CEA: Carcinoembryonic antigen
CT: Computed tomography
6MWT: 6-Minute walk test
MDT: Master’s double two-step test
METs: Metabolic equivalents of task
NSCLC: Non-small cell lung cancer
PET: Positron emission tomography
CCI: Charlson comorbidity index
